# GERONTECHNOLOGY PERCEPTIONS AND POTENTIAL ROLE OF VR/AR IN OPTIMAL AGING

**DOI:** 10.1093/geroni/igz038.894

**Published:** 2019-11-08

**Authors:** Jennifer Margrett, Wally Boot, Neil Charness, Christopher Hertzog, Mack Shelley, Balaji Narasimhan

**Affiliations:** 1 Iowa State University, Ames, Iowa, United States; 2 Florida State University, Tallahassee, Florida, United States; 3 Georgia Institute of Technology, Atlanta, Georgia, United States

## Abstract

Technology presents opportunities to optimize whole person wellness and functioning. To understand tech readiness and the potential role of virtual (VR) and augmented reality (AR) to support optimal aging, we surveyed 604 participants from the nationally representative RAND American Life Panel. Participant age ranged from 50-90+, 51.5% were female, and 50% reported bachelor’s education or higher. Overall, 8% of the sample identified as Hispanic, with 15% of individuals also identifying as Black, Asian, or Asian Indian or Alaskan Native. Males reported greater optimism and technology innovation and adults aged 50-64 were the most optimistic. Overall, 80% of the sample reported VR familiarity compared to 33% AR. Regarding future needs, 75% of the participants expressed specific concerns about future ADL ability. Almost half of the respondents indicated willingness to use VR and AR to maintain or improve functioning with age and increased personalization of optimal aging emerged as a significant predictor.

